# Comparative Effects of Different Nutritional Supplements on Inflammation, Nutritional Status, and Clinical Outcomes in Colorectal Cancer Patients: A Systematic Review and Network Meta-Analysis

**DOI:** 10.3390/nu15122772

**Published:** 2023-06-16

**Authors:** Jiayi Ye, Yanjie Hu, Xinrong Chen, Chengting Chang, Ka Li

**Affiliations:** West China Hospital, Sichuan University/West China School of Nursing, Sichuan University, Chengdu 610041, China

**Keywords:** nutritional supplements, colorectal cancer, inflammation, nutrition, network meta-analysis

## Abstract

Nutritional supplements have been widely used in colorectal cancer (CRC) patients. The aim of this network meta-analysis (NMA) was to compare the effects of different nutritional supplements on inflammation, nutritional status, and clinical outcomes in CRC patients. Four electronic databases were searched until December 2022. Randomized controlled trials (RCTs) comparing nutritional supplements of omega-3 fatty acids (omega-3), arginine, vitamin D, glutamine, probiotics, or their combinations with placebo or standard treatment were selected. The outcomes were inflammatory indicators, nutritional indicators, and clinical outcomes. A random-effects Bayesian NMA was performed to rank the effect of each supplement. In total, 34 studies involving 2841 participants were included. Glutamine was superior in decreasing tumor necrosis factor-α (MD −25.2; 95% CrI [−32.62, −17.95]), whereas combined omega-3 and arginine supplementation was more effective in decreasing interleukin-6 (MD −61.41; 95% CrI [−97.85, −24.85]). No nutritional supplements significantly maintained nutritional indicators in CRC patients. Regarding clinical outcomes, glutamine ranked highest in reducing the length of hospital stay (MD −3.71; 95% CrI [−5.89, −1.72]) and the incidence of wound infections (RR 0.12; 95% CrI [0, 0.85]), and probiotics were rated as best in reducing the incidence of pneumonia (RR 0.38; 95% CrI [0.15, 0.81]). Future well-designed RCTs are needed to further confirm these findings.

## 1. Introduction

Globally, colorectal cancer (CRC) is the third most frequently diagnosed cancer and the second leading cause of cancer-related deaths. In 2020, approximately 1.9 million new cases of CRC emerged and 935,000 deaths occurred, accounting for approximately one-tenth of cancer cases and deaths [1]. Standard conventional treatments for CRC include surgery, chemotherapy, and radiation therapy. Depending on the localization and progression of the tumor, these treatments can be used in combination [2,3,4,5]. Due to long-term tumor consumption, inadequate nutritional intake, as well as stress responses and metabolic disorders caused by surgical trauma, chemotherapy, or radiotherapy, patients are prone to chronic inflammation, malnutrition, and complications [6,7,8,9]. Inflammation is a driving factor contributing to cancer progression, invasion, metastasis, and adverse clinical characteristics, such as malnutrition, weight loss, and fever, which can severely affect patients’ prognosis [10]. The prevalence of malnutrition in CRC patients ranges from 20 to 37% [11], which can cause treatment outcomes to decrease and negatively impact patient prognosis and long-term quality of life [6,12,13,14,15]. Furthermore, inflammation and malnutrition may be the causal factors for poor clinical outcomes such as wound infections (WI), anastomotic leaks (AL), and pneumonia in CRC patients [10,16]. The incidence of adverse clinical outcomes raises patients’ medical expenses and impedes their recovery process [17,18]. Therefore, it is critical to find effective interventions to reduce inflammation and improve the nutritional status and clinical outcomes of CRC patients.

Several clinical studies have demonstrated the potency of nutritional supplements, such as omega-3 fatty acids (omega-3), arginine, vitamin D, glutamine, and probiotics, as an effective intervention in decreasing inflammation and enhancing the nutritional status, as well as the overall clinical outcomes of CRC patients [8,19,20,21,22,23,24,25,26,27,28]. For instance, glutamine supplementation was discovered to modulate the immunometabolic response of CRC patients and lower their susceptibility to infections [22,23]. CRC patients consuming vitamin D and omega-3 also showed low inflammation and better nutritional status [19]. Furthermore, it has been observed that probiotic supplementation modulated gut microbiota in CRC patients, reduced bacterial translocation and inflammatory cytokine release, and improved clinical outcomes in patients [24].

To date, multiple systematic reviews and meta-analyses have reported similar results in which nutritional supplements have been shown to improve inflammation, nutritional status, and clinical outcomes in CRC patients [8,25,26]. However, the comparative effects of different nutritional supplements in CRC patients have not been estimated. Network meta-analysis (NMA) allows for the simultaneous comparison of the effect of multiple interventions, as direct comparisons between these nutritional supplements are lacking [29]. Moreover, NMA can also rank interventions based on various outcomes, aiding health professionals and clinicians in evidence-based decision-making. Much prior research suggests that omega-3, arginine, vitamin D, glutamine, and probiotics are the most effective and widely studied nutritional supplements for reducing inflammation and improving nutritional status and clinical outcomes in CRC patients [8,19,20,21,22,23,24,25,26,27,28]. Therefore, in this study, we selected these nutritional supplements, aiming to compare and rank the effects of these nutritional supplements on inflammation, nutritional status, and clinical outcomes in CRC patients, as well as provide evidence-based data on nutritional supplementation for CRC patients.

## 2. Method

### 2.1. Design and Registration

We conducted the NMA following the Preferred Reporting Items for Systematic Reviews and Meta-Analyses (PRISMA) statement [30], and this study has been registered in the International Prospective Register of Systematic Reviews (PROSPERO; identifier CRD42023392142).

### 2.2. Inclusion and Exclusion Criteria

Inclusion criteria were as follows: (1) Patients diagnosed with CRC, colon cancer, or rectal cancer of any age. (2) Patients in the intervention group received at least one of the following nutritional supplements (in any form, dose, or duration): glutamine, arginine, omega-3, probiotics, vitamin D, or a combination of these supplements. These nutritional supplements are the most well-studied in CRC patients. (3) Patients in the control group received either placebo or standard treatment. If patients in both intervention groups and control groups received general adjuvant therapy at the same time, the adjuvant therapy should be the same. (4) Studies reported at least one of the following outcomes: inflammatory indicators such as C-reactive protein (CRP), tumor necrosis factor-α (TNF-α), and interleukin-6 (IL-6); nutritional indicators such as albumin (Alb), weight, and body mass index (BMI); clinical outcomes such as length of hospital stay (LOH), the incidence of urinary tract infections (UTI), the incidence of WI, the incidence of AL, and the incidence of pneumonia. (5) The study design was a randomized controlled trial (RCT). (6) The language of the studies was limited to English.

Exclusion criteria were as follows: (1) Patients with cancer types other than CRC. (2) Studies with unclear diagnostic and efficacy criteria. (3) Studies that were cohort studies, review articles, case reports, descriptive studies, opinion articles, or abstracts. (4) Studies with incomplete or erroneous data that could not be merged.

### 2.3. Search Methods

Two researchers (Y.J.Y. and H.Y.J.) independently performed an extensive search on PubMed, Embase, the Cochrane Central Register of Controlled Trials, and Web of Science up to 25 December 2022, without restrictions in terms of the document type, date/time, and publication status. Medical Subject Headings (MeSH) and the free words of the keywords, including all known spellings of “colorectal cancer”, “glutamine”, “arginine”, “omega-3 fatty acids”, “probiotics”, and “vitamin D” were applied for document retrieval (for searching strategies, see Supplementary Table S1).

### 2.4. Study Selection

According to our predefined inclusion and exclusion criteria mentioned above, two researchers (Y.J.Y. and H.Y.J.) independently conducted the study selection. First, all potentially relevant studies were imported into EndNote 20 to remove repetitive studies. Then, we screened titles and abstracts to exclude ineligible studies. Finally, the full texts were further screened. Disagreements were resolved through discussion or negotiation with a third researcher (C.X.R.).

### 2.5. Data Extraction and Quality Assessment

The Cochrane data extraction form was used to extract the following data and information: (1) basic information such as title, name of the first author, and year of publication; (2) basic characteristics of the research subjects, including age, gender, the numbers of cases in each group, and diagnosis criteria; (3) details about the intervention, including specific measures and timing; (4) outcome indicators such as the levels of CRP, TNF-α, IL-6, Alb, weight, BMI, and LOH, the incidence of UTI, WI, AL, and pneumonia were extracted for the baseline and post-treatment timepoints; (5) key elements of risk assessment for bias. One researcher (Y.J.Y.) extracted data, which was then confirmed for accuracy by another researcher (H.Y.J.).

The methodological quality of each included study was assessed using the modified Jadad scale [31]. Two researchers (Y.J.Y. and H.Y.J.) independently scored the following items: generation of random sequences, allocation concealment, intervention blinding of participants and investigators, and incomplete outcome data. The modified Jadad scale scores range from 0 to 7. Studies with scores >3 were considered high quality, while studies with scores ≤3 were classified as low quality. The above scores were cross-checked and solutions were discussed with the third researcher (C.X.R.) if there were any discrepancies.

### 2.6. Data Synthesis and Statistical Analysis

Statistical models based on the Bayesian framework were constructed using the JAGS software (gemtc 0.8–2 and rjags 4–10 package) in R (version 4.1.2) (Rstudio, Boston, MA, USA). The mean difference (MD) with a 95% credible interval (CrI) was calculated for continuous data to determine the effect size. A pooled risk ratio (RR) with a 95% CrI was calculated for categorical data. Random-effect models for all NMA were employed because the trials included were clinically heterogeneous (different countries, doses of nutritional supplements, routes of supplementation, duration of supplementation, and anti-cancer treatment). Four Markov chains were set for each outcome, and each chain produced 50,000 iterations, with 20,000 iterations discarded as a burn-in period. The convergence of iterations was assessed with plots and the Gelman–Rubin–Brooks statistic [32]. We used the surface under the cumulative rank curve (SUCRA) to estimate the relative rank of different nutritional supplements for each outcome of interest [33]. The higher the SUCRA value, the higher the intervention in the rank [33]. In addition, the consistency model and inconsistency model were compared using the deviation information criterion (DIC). If the difference in DIC was less than 5 points, consistency was considered good and consistency modeling was used [34]. Heterogeneity was assessed using the I^2^ statistic. I^2^ values below 25% were considered low heterogeneity, those of 25 to 75% were considered moderate heterogeneity, and those higher than 75% were considered high heterogeneity [35]. Comparison-adjusted funnel plots were used to test for publication bias. Network plots and comparison-adjusted funnel plots of NMA were drawn by Stata (version 17.0) (StataCorp, College Station, Texas, USA).

## 3. Results

### 3.1. Search Outcomes

**Figure 1:**
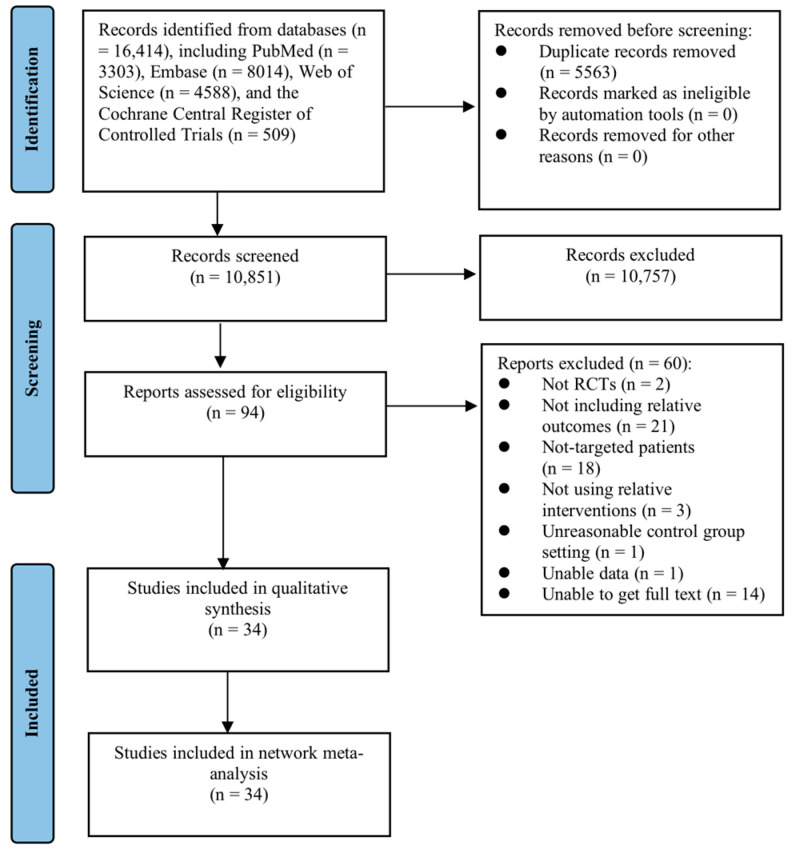
PRISMA flow diagram for search and selection of eligible studies included in the network meta-analysis.

The detailed procedure of study selection is shown as a PRISMA flow diagram in Figure 1. Initially, a total of 16,414 potentially relevant studies were identified from the four electronic databases mentioned above. After removing 5563 repetitive studies, the title and/or abstract of each study were screened based on the inclusion and exclusion criteria. Subsequently, 10,757 studies were excluded. The remaining 94 studies were further assessed for eligibility by examining their full texts, out of which 60 studies were excluded as they did not meet the criteria of RCTs, did not target the desired patients, and lacked data (details see Figure 1). Ultimately, 34 studies were deemed suitable and included in the NMA.

### 3.2. Characteristics of Included Studies

The characteristics and details of each included study in our NMA are presented in Table 1. Of the 34 eligible studies published from 1998 to 2022, 20 were conducted in Asia [19,20,22,36,37,38,39,40,41,42,43,44,45,46,47,48,49,50,51,52], 10 in Europe [27,53,54,55,56,57,58,59,60,61], and 4 in South America [24,62,63,64]. Overall, 2841 CRC patients participated in the 34 RCTs, with study sample sizes ranging from 11 to 362 and mean ages ranging from 50 to 72 years old. Among them, 25 studies recruited patients with CRC surgery [20,22,24,27,36,37,40,41,42,43,44,45,47,48,49,50,51,52,53,54,55,56,57,61,63], 6 studies recruited patients with CRC chemotherapy [19,38,39,60,62,64], and 3 studies recruited patients with CRC chemoradiotherapy [46,58,59]. During the time of nutritional interventions, 7 studies intervened at the preoperative time point [24,27,40,41,43,47,57], 12 studies intervened during the perioperative period [22,42,44,45,48,49,50,51,53,54,56,63], 6 studies intervened postoperatively [20,36,37,52,55,61], 6 studies intervened during chemotherapy [19,38,39,60,62,64], and 3 studies intervened during chemoradiotherapy [46,58,59]. Depending on the means of nutritional intervention, 8 studies were administrated through parenteral nutrition [22,36,37,51,52,53,60,61], 24 studies were administrated through oral nutrition [19,20,24,38,39,40,41,42,43,44,45,46,47,48,49,50,54,55,57,58,59,62,63,64], and 2 studies were administrated through oral and enteral nutrition [27,56]. These studies evaluated a total of 9 nutritional supplements: glutamine (*n* = 7) [22,37,51,58,59,60,61], arginine (*n* = 1) [57], omega-3 (*n* = 9) [19,36,37,38,52,53,54,62,64], probiotics (*n* = 16) [20,24,40,41,42,43,44,45,46,47,48,49,50,55,56,63], vitamin D (*n* = 1) [19], combined with omega-3 and arginine (omega-3 + arginine) (*n* = 1) [27], combined with omega-3 and vitamin D (omega-3 + vitamin D) (*n* = 1) [19], combined with omega-3 and probiotics (omega-3 + probiotics) (*n* = 1) [39], and combined with omega-3 and glutamine (omega-3 + glutamine) (*n* = 1) [37]. As for the study design, all included studies were RCTs, among which 32 studies were two-arm studies [20,22,24,27,36,38,39,40,41,42,43,44,45,46,47,48,49,50,51,52,53,54,55,56,57,58,59,60,61,62,63,64] and 2 were four-arm studies [19,37].

### 3.3. Quality Assessment

The Jadad quality assessment scale showed that all 34 studies were of high quality (Jadad score > 3) (Table 1).

### 3.4. Network Meta-Analysis

#### 3.4.1. Inflammatory Indicators

##### Tumor Necrosis Factor-α

**Figure 2:**
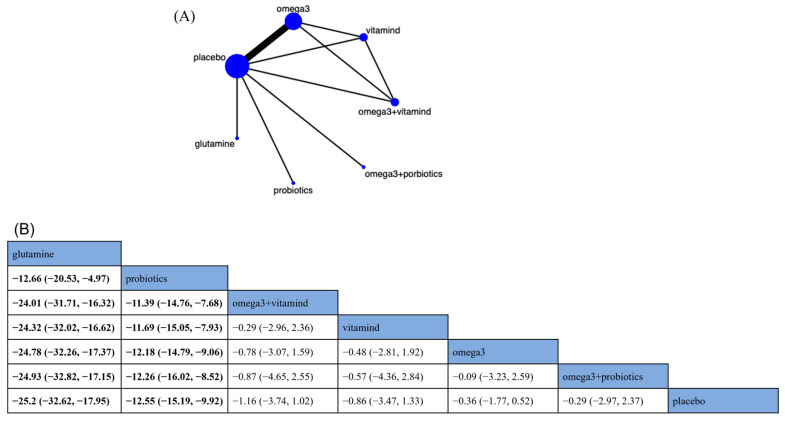
Network plot and results of network meta-analysis. (**A**) Network plot for tumor necrosis factor-α (TNF-α, pg/mL). (**B**) Relative effects of different nutritional supplements on TNF-α. Notes: estimates are presented as mean difference (MD) and 95% credible intervals (CrI; in parentheses). Comparisons between treatments should be read from left to right. The estimate of supplementation effectiveness is located at the intersection of the column-defining supplementation and the row-defining supplementation. Significant results are presented in bold. Combined with omega-3 fatty acids and probiotics: omega3 + probiotics; combined with omega-3 fatty acids and vitamin D: omega3 + vitamind; omega-3 fatty acids: omega3; vitamin D: vitamind.

Overall, eight RCTs assessed the effects of six nutritional supplements on TNF-α (Figure 2A). The main findings of the NMA are shown in Figure 2B. Compared with placebo, glutamine (MD −25.2; 95% CrI [−32.62, −17.95]) and probiotics (MD −12.55; 95% CrI [−15.19, −9.92]) significantly reduced TNF-α levels in CRC patients (Figure 2B). Based on SUCRA, glutamine was the best nutritional supplement to reduce TNF-α (SUCRA = 99.9%) (Supplementary Table S5).

##### Interleukin-6

**Figure 3:**
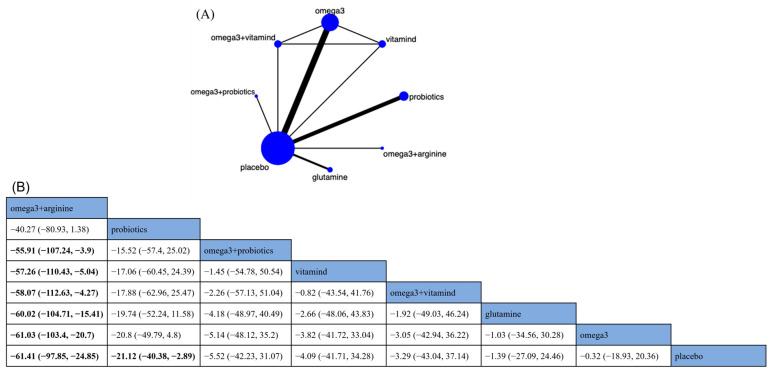
Network plot and results of network meta-analysis. (**A**) Network plot for interleukin-6 (IL-6, pg/mL). (**B**) Relative effects of different nutritional supplements on IL-6. Notes: estimates are presented as mean difference (MD) and 95% credible intervals (CrI; in parentheses). Comparisons between treatments should be read from left to right. The estimate of supplementation effectiveness is located at the intersection of the column-defining supplementation and the row-defining supplementation. Significant results are presented in bold. Combined with omega-3 fatty acids and arginine: omega3 + arginine; combined with omega-3 fatty acids and probiotics: omega3 + probiotics; combined with omega-3 fatty acids and vitamin D: omega-3 + vitamind; omega-3 fatty acids: omega3; vitamin D: vitamind.

In total, fourteen RCTs and seven nutritional supplements were analyzed for their effects on IL-6 (Figure 3A). Compared with the placebo, omega-3 + arginine (MD −61.41; 95% CrI [−97.85, −24.85]) and probiotics (MD −21.12; 95% CrI [−40.38, −2.89])] significantly reduced IL-6 levels in CRC patients (Figure 3B). The remaining nutritional supplements also reduced IL-6 levels in CRC patients, but the difference did not reach significance (Figure 3B). The ranking based on SUCRA showed that omega-3 + arginine was the best choice to reduce IL-6 levels (SUCRA = 98.6%) (Supplementary Table S5).

##### C-reactive Protein

Seven nutritional supplements (arginine, probiotics, omega-3 + probiotics, omega-3 + vitamin D, omega-3, vitamin D, and glutamine) from nine RCTs were included in the analysis of CRP (Supplementary Figure S1). It was found that none of the nutritional supplements significantly reduced CRP levels compared with the placebo (Supplementary Table S2**)**. There were also no significant differences in the comparative effects among these nutritional supplements (for details please see Supplementary Table S2). According to the SUCRA ranking, vitamin D may be the best nutritional supplement to reduce CRP (SUCRA = 87.2%) (Supplementary Table S5).

#### 3.4.2. Nutritional Indicators

Nutritional indicators were evaluated, including Alb, weight, and BMI levels. The network plot was shown in Supplementary Figure S2. In total, the relative effects of four nutritional supplements (omega-3, omega-3 + vitamin D, vitamin D, and probiotics) on Alb levels were envaulted from six RCTs, the relative effects of six nutritional supplements (omega-3, vitamin D, omega-3 + vitamin D, omega-3 + probiotics, probiotics, and glutamine) on the levels of weight were evaluated from six RCTs, and the relative effects of five nutritional supplements (probiotics, omega-3 + probiotics, vitamin D, omega-3, and omega-3 + vitamin D) from five RCTs on BMI levels were analyzed (Supplementary Figure S2). These nutritional supplementation interventions were administered prior to, or in conjunction with, treatment (details see Table 1). The NMA showed that most nutritional supplements reduced the decline in Alb, weight, and BMI levels during patient treatment relative to placebo, but the differences were not statistically significant (Supplementary Table S3). The SUCRA ranking showed that omega-3 + vitamin D may be the best choice to maintain the Alb (SUCRA = 76.9%), weight (SUCRA = 66.6%), and BMI of patients during treatment (SUCRA = 63.6%) (Supplementary Table S6).

#### 3.4.3. Clinical Outcomes

##### Length of Hospital Stay

**Figure 4:**
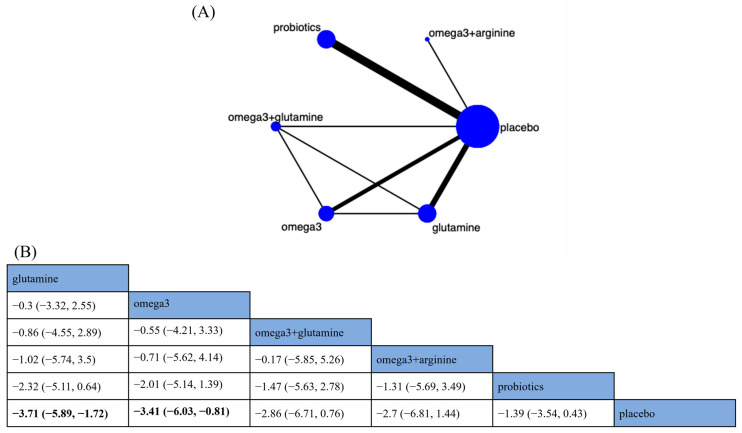
Network plot and results of network meta-analysis. (**A**) Network plot for the length of hospital stay (LOH). (**B**) Relative effects of different nutritional supplements on the LOH. Notes: estimates are presented as mean difference (MD) and 95% credible intervals (CrI; in parentheses). Comparisons between treatments should be read from left to right. The estimate of supplementation effectiveness is located at the intersection of the column-defining supplementation and the row-defining supplementation. Significant results are presented in bold. Combined with omega-3 fatty acids and arginine: omega3 + arginine; combined with omega-3 fatty acids and glutamine: omega3 + glutamine; omega-3 fatty acids: omega3.

Five nutritional supplements across thirteen RCTs were analyzed using NMA to evaluate the effects of nutritional supplements on the LOH in CRC patients (Figure 4A). The NMA showed that glutamine (MD −3.71; 95% CrI [−5.89, −1.72]) and omega-3 (MD −3.41; 95% CrI [−6.03, −0.81]) significantly reduced the LOH in CRC patients compared with placebo (Figure 4B). In terms of SUCRA ranking, glutamine was the best nutritional supplement to reduce the LOH (SUCRA = 78.7%) (Supplementary Table S7).

##### Urinary Tract Infections

The network plot of UTIs is shown in Supplementary Figure S3A, which includes a total of four nutritional supplements (probiotics, glutamine, omega-3, and omega-3 + arginine) from nine RCTs. According to the results of the NMA, none of the supplements significantly reduced the incidence of UTI, and there were no significant differences in the relative effects among the various supplements (Supplementary Table S4A). Based on SUCRA, probiotics may be the best nutritional supplement to reduce the incidence of UTI (SUCRA = 83.5%) (Supplementary Table S7).

##### Wound Infections

**Figure 5:**
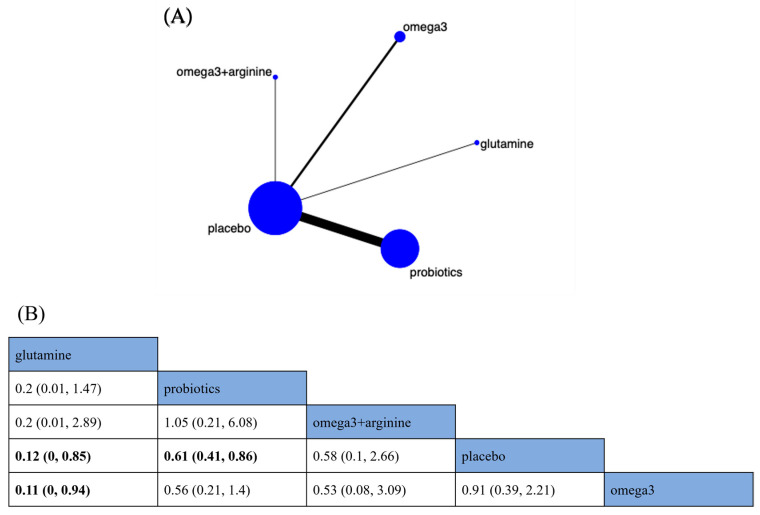
Network plot and results of network meta-analysis. (**A**) Network plot for the incidence of wound infections (WI). (**B**) Relative effects of different nutritional supplements on the incidence of WI. Notes: estimates are presented as risk ratio (RR) and 95% credible intervals (CrI; in parentheses). Comparisons between treatments should be read from left to right. The estimate of supplementation effectiveness is located at the intersection of the column-defining supplementation and the row-defining supplementation. Significant results are presented in bold. Combined with omega-3 fatty acids and arginine: omega3 + arginine; omega-3 fatty acids: omega3.

The four nutritional supplements from the seventeen RCTs provided data to compare their relative effects on the incidence of WIs (Figure 5A). Compared with placebo, glutamine and probiotics reduced the risk of WIs by 88% (RR 0.12; 95% CrI [0, 0.85]) and 39% (RR 0.61; 95% CrI [0.41, 0.86]), respectively (Figure 5B). In addition, a similar advantage exists for glutamine compared to omega-3 (RR 0.11; 95% CrI [0, 0.94]) in reducing the risk of WIs (Figure 5B). According to SUCRA, glutamine was the best nutritional supplement to reduce the incidence of WIs (SUCRA = 94.5%) (Supplementary Table S7).

##### Anastomotic Leaks

There were four nutritional supplements (omega-3 + arginine, omega-3, glutamine, and probiotics) from twelve RCTs included in the analysis of Als (Supplementary Figure S3B). The NMA showed that no nutritional supplements significantly reduced the incidence of ALs (Supplementary Table S4B). Moreover, the relative effects between different nutritional supplements were also not statistically significant (Supplementary Table S4B). Based on SUCRA, glutamine may be the best choice to reduce the incidence of ALs (SUCRA = 84.1%) (Supplementary Table S7).

##### Pneumonia

**Figure 6:**
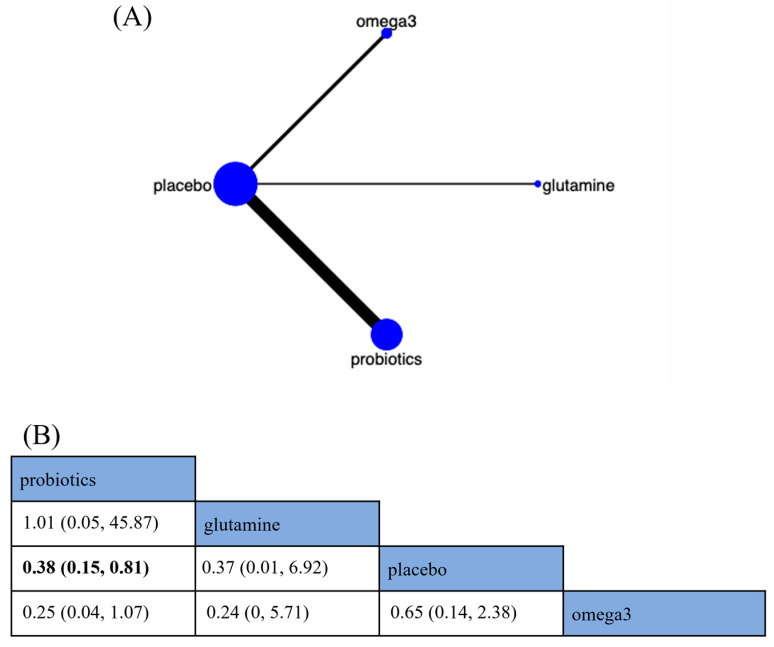
Network plot and results of network meta-analysis. (**A**) Network plot for the incidence of pneumonia. (**B**) Relative effects of different nutritional supplements on the incidence of pneumonia. Notes: estimates are presented as risk ratio (RR) and 95% credible intervals (CrI; in parentheses). Comparisons between treatments should be read from left to right. The estimate of supplementation effectiveness is located at the intersection of the column-defining supplementation and the row-defining supplementation. Significant results are presented in bold. Omega-3 fatty acids: omega3.

The incidence of pneumonia was reported in ten RCTs and involved three different nutritional supplements (Figure 6A). The analysis revealed that probiotics reduced the risk of pneumonia by 62% (RR 0.38; 95% CrI [0.15, 0.81]) compared to placebo (Figure 6B). The relative comparisons between different nutritional supplements were not statistically significant (Figure 6B). According to SUCRA, probiotics were the best nutritional supplement to reduce the incidence of pneumonia (SUCRA = 82.0%) (Supplementary Table S7).

### 3.5. Consistency and Publication Bias Assessment

The consistency model and inconsistency model were compared using DIC. Good consistency with DIC was indicated by changes less than five for all closed-loop models present. In terms of publication bias assessment, there was no evidence of publication bias in the comparison-adjusted funnel plots (Supplementary Figure S4).

## 4. Discussion

We conducted a comprehensive search of relevant publications and analyzed all available evidence from 34 RCTs with NMA to compare the effects of various nutritional supplements on inflammation, nutritional status, and clinical outcomes in CRC patients. Regarding inflammatory indicators, glutamine was rated as the most effective in reducing TNF-α levels, while omega-3 + arginine had the best potency to reduce IL-6 levels. However, none of the nutritional supplements significantly maintained the nutritional indicators of CRC patients. Regarding clinical outcomes, glutamine was the most effective nutritional supplement to reduce the LOH and the incidence of WI, whereas probiotics were rated as best in lowering the incidence of pneumonia in CRC patients.

Based on NMA, it was found that glutamine was significantly effective in reducing TNF-α levels in CRC patients, whereas omega-3 + arginine significantly inhibited IL-6 expression in CRC patients. Inflammation is a crucial factor in the proliferation and invasion of CRC, and severe inflammation can lead to cancer progression and worsen the prognosis of patients [10]. Previous systematic reviews and meta-analyses have shown that supplements such as glutamine, probiotics, omega-3, and arginine can attenuate inflammatory responses [65,66,67], coinciding with our results. The present study further confirms that optimal nutritional supplement intake may be used to suppress the expression of different inflammatory cytokines in CRC patients. As an immune nutrient, glutamine exhibits important regulatory effects on the proliferation and function of lymphocytes, macrophages, and neutrophils [68]. Furthermore, it can also reduce excessive inflammatory responses [68]. Taniguchi et al. showed that glutamine supplementation in colitis-associated CRC mice significantly reduced TNF-α levels [69]. Additionally, omega-3 can also decrease the expression of nuclear factor- κB (NF-κB) and peroxisome proliferator-activated receptors (PPARs), thereby suppressing the production of inflammatory cytokines [70]. Omega-3 can also be converted into anti-inflammatory lipid mediators that bind to specific receptors on immune cells to inhibit the production of proinflammatory cytokines [71]. Arginine can also suppress excessive inflammatory responses through the production of nitric oxide and immunomodulators such as resolvin and protectin [72,73]. Consistent with previous studies, we found that nutritional supplements did not substantially reduce CRP levels in CRC patients [15,73]. However, these findings should be interpreted cautiously due to the limited number of available trials. Of note, our study provides a rationale for the use of nutritional supplementation regimens with glutamine and omega-3 + arginine in patients with CRC. These supplements may improve the prognosis of CRC patients by reducing their excessive inflammatory response, which provides new insight for the future adjunctive treatment of CRC patients.

Based on our study, none of the nutritional supplements had a significant effect on maintaining the Alb, weight, and BMI levels of CRC patients during treatment. A meta-analysis study showed that perioperative omega-3 supplementation had no significant effect on maintaining weight, BMI, and Alb levels in patients with gastrointestinal cancer [74]. Furthermore, Brown et al. [75] reported that vitamin D supplementation during chemotherapy did not significantly improve weight and BMI in CRC patients. The results of the above study were consistent with our analysis. However, there were diverse findings, such as those by Yue et al. [8]. The results of a meta-analysis by Yue et al. showed that perioperative omega-3 supplementation significantly increased Alb levels in CRC patients [8]. This discrepancy may be attributed to heterogeneity in the study populations, including the baseline weight loss, types of anticancer treatment, and tolerance to the anticancer treatment. Furthermore, Alb, although used as a surrogate for nutritional status, can be influenced by other factors such as inflammation, renal function, and hydration status [76]. Although the results of our study did not reach significance, it is noteworthy that nutritional supplementation interventions before or during treatment were shown to be associated with maintaining the nutritional status of CRC patients during treatment. Importantly, maintaining the nutritional status of CRC patients during treatment can help to reduce the toxicity of the treatment and decrease the incidence of complications, thereby improving the prognosis and quality of life of patients [6,12]. Moreover, our NMA showed that omega-3 + vitamin D ranked highest in maintaining Alb, weight, and BMI levels in CRC patients during treatment. Malnutrition in CRC patients is typically associated with cancer-induced systemic inflammation, leading to anabolic resistance and muscle loss [6]. Omega-3 and vitamin D can stimulate immune cells in specific ways, such as by enhancing response function, maintaining a normal and moderate immune response, regulating cytokine production and release, and reducing harmful or excessive inflammatory responses, thereby maintaining the nutritional status of CRC patients [77,78,79,80]. The above evidence indicates that omega-3 + vitamin D has the most potential to be used to maintain the nutritional status of CRC patients during treatment.

Despite advancements in surgical procedures and perioperative care, CRC patients still experience adverse postoperative clinical outcomes after surgery. These poor outcomes not only increase medical expenses but also reduce the quality of life of patients [81,82]. Previous meta-analysis results showed that glutamine and omega-3 supplementation can significantly reduce LOH in CRC patients [28,83]. Our NMA confirmed these results and demonstrated that glutamine was the most effective nutritional supplement for reducing LOH in CRC patients. Glutamine is an immune nutrient that can improve the immune response of CRC patients, decrease the incidence of infectious complications, and subsequently reduce the LOH of CRC patients [68,84]. Similar to a previous study, we also found that glutamine was the most effective nutritional supplement to reduce the incidence of WI in CRC patients [28]. This may be related to the anti-inflammatory and healing-promoting effects of glutamine. Glutamine not only inhibits the excessive inflammatory response by regulating the body’s immune response but also promotes nitric oxide synthesis, which modulates cell proliferation, collagen formation, and wound contraction, thereby facilitating wound healing [68,85,86,87]. However, the extent of the effect of glutamine on the incidence of WI in CRC patients in the present study should be interpreted with caution, as only one trial was available. Moreover, our NMA showed that probiotics performed best in decreasing the incidence of pneumonia in CRC patients. This result aligns with a meta-analysis study by Araújo et al. [26]. The mechanism by which probiotics reduce the risk of postoperative pneumonia in CRC remains unclear. However, some proposed mechanisms include increasing the number of beneficial bacteria in the gut microbiota and maintaining the balance of the gut microbiota [88]. The balance of microbiota plays an essential role in the development and function of the immune system, both locally and systemically [89]. Secondly, probiotic supplementation significantly increases the levels of short-chain fatty acids, which promote anti-inflammatory mechanisms to maintain intestinal and systemic homeostasis [89]. Third, probiotic supplementation could maintain the intestinal epithelial barrier. Probiotics stimulate tight junction proteins in the intestinal epithelial cell, which promotes mucin secretion, increases the protective layer, prevents pathogenic bacteria from adhering to intestinal epithelial cells, and consequently reduces the translocation of pathogenic bacteria [90]. Finally, probiotics can regulate innate and adaptive immune responses and reduce systemic inflammatory responses [91]. These changes suggest that probiotic supplementation can have a beneficial effect on reducing the occurrence of pneumonia. Nonetheless, this study did not find that nutritional supplements significantly improved UTI incidence and AL incidence in CRC patients, which may be due to differences in the study population, study design, and protocol (the variability in the nutritional supplement routes, doses, start time, and duration). Based on our research findings, glutamine and probiotics can be used in patients undergoing CRC surgery to improve their clinical outcomes.

## 5. Strengths and Limitations

To our knowledge, this is the first NMA to compare the effects of different nutritional supplements on inflammation, nutritional status, and clinical outcomes in CRC patients, and to rank all supplements based on these factors. This study provides valuable information when deciding on the best nutritional intervention for CRC patients. Nevertheless, this NMA has several limitations that should be acknowledged. Firstly, the small sample sizes and the limited number of studies may affect the accuracy and applicability of our results. Secondly, the varying treatment of patients, the formula, dose, route, and time of administration of nutrition supplements among the studies included may result in heterogeneity. Unfortunately, subgroup analyses could not be performed due to the limited number of studies. Lastly, our study was limited to research published in English, introducing selectivity bias. Hence, more high-quality RCTs are required to confirm our findings.

## 6. Conclusions

There is no single nutritional supplement that works optimally for all indicators in CRC patients. Glutamine was found to be the most effective in lowering TNF-α levels, shortening LOH, and reducing the incidence of WI in CRC patients. Omega-3 + arginine performed optimally in reducing IL-6 levels in CRC patients. Probiotics performed best in reducing the incidence of pneumonia in CRC patients. Due to some limitations of existing clinical studies and evidence, future studies should focus on larger sample sizes, longer follow-ups, and more rigorous study designs to confirm these findings.

## Figures and Tables

**Table 1:** Main characteristics of the studies included in the network meta-analysis.

Author (Year)	Nationality	Participant Types	Sample Size (*n*)		Age (Years)		Intervention	Route	Outcomes	Jadad Score
			Treatment	Control	Treatment	Control				
Aliyazicioglu et al. (2013) [37]	Turkey	CRC surgery patients	8/8/10	10	58.0 ± 15.6/ 63.9 ± 17.2/ 59.9 ± 15.1	56.3 ± 14.3	glutamine/omega3 fatty acid/ glutamine + omega 3 fatty acid/glutamine:0.3–0.4 g/kg/d, omega 3 fatty acid: 0.1–0.2 g/kg/d, 2 postoperative days to 7 postoperative days	PN	g	4
Bakker et al. (2020) [53]	Netherlands	CRC surgery patients	18	23	68.2475 ± 8.849	68.6415 ± 8.6919	omega 3 fatty acid (fish oil)/0.2 g/kg/d, 1 preoperative day and 1 postoperative day	PN	a, c, h, i, j, k	6
Sorensen et al. (2014) [54]	Denmark	CRC surgery patient	74	74	69 ± 11	71 ± 10	omega 3 fatty acid (EPA:2.0 g/d and DHA:1.0 g/d)/400 mL/d, 7 preoperative days and 7 postoperative days	Oral	h, i, j, k	7
Liang et al. (2008) [36]	China	CRC surgery patients	20	21	55.80 ± 10.10	59.19 ± 10.61	omega 3 fatty acid/0.2 g/kg/d, 7 postoperative days	PN	b, c, g	7
Esfahani et al. (2016) [38]	Iran	CRC chemotherapy patients	36	35	54.14 ± 10.53	53.40 ± 15.70	omega 3 fatty acid (54% DHA, 10% EPA)/1920 mg/d, 1 month	Oral	b, c	7
Mocellin et al. (2013) [62]	Brazil	CRC chemotherapy patients	6	5	55.2 ± 7.7	53.6 ± 12.9	omega 3 fatty acid (90 mg EPA and 60 mg DHA)/2 g/d, 9 weeks	Oral	a, b, d, e, f	4
Haidari et al. (2020) [19]	Iran	CRC chemotherapy patients	20/21/20	20	56.75 ± 10.60/ 56.90 ± 12.45/ 57.15 ± 10.17	59.90 ± 8.75	omega 3 fatty acid (54 mg EPA, 250 mg DHA, 26 mg other omega-3 fatty acids)/ Vitamin D/ omega 3 fatty acid (54 mg EPA, 250 mg DHA, 26 mg other omega-3 fatty acids) + vitamin D/vitamin D: 50,000 IU soft gel/w, omega3 fatty acid: 660 mg/d, 8 weeks	Oral	a, b, c, d, e, f	4
Golkhalkhali et al. (2017) [39]	Malaysia	CRC chemotherapy patients	70	70	≤56:20 57–66: 22 ≥67:23	≤56:28 57–66:19 ≥67:19	omega 3 fatty acid (700 mg EPA) + probiotics (L. acidophilus, Lactobacillus lactis, Bififidobacterium bififidum, Bififidobacterium longum, Bififidobacterium infantis)/omega 3 fatty acid: 2 g/d + probiotics:2 sachets/d, 8 weeks	Oral	a, b, c, e, f	7
Bajramagic et al. (2019) [55]	Sarajevo	CRC surgery patients	39	39	NR	NR	probiotics (Lactobacillus acidophilus, Lactobacillus casei, Lactobacillus plantarum, Lactobacillus rhamnosus, Bifidobacterium lactis, Bifidobacterium bifidum, Bifidobacterium breve, Streptococcusthermophilus)/4 capsules/d for 30 days, 1 capsule/d for 2 weeks in each month to 1 year	Oral	i, j	4
Tan et al. (2016) [40]	Malaysia	CRC surgery patients	20	20	64.3 ± 14.5	68.4 ± 11.9	probiotics (Lactobacillus acidophilus, Lactobacillus lactis, Bififidobacterium bififidum, Bififidobacterium longum, and Bififidobacterium infantis)/2 sachets/d, 7 preoperative days	Oral	g, i, j, k	6
Zhang et al. (2012) [41]	China	CRC surgery patients	30	30	67.14 ± 10.29	62.09 ± 8.82	probiotics (B longum, L acidophilus, and Enterococcus faecalis)/0.63 g/d, 5 preoperative days to 3 preoperative days	Oral	c, i, j, k	5
Flesch et al. (2017) [63]	Brazil	CRC surgery patients	49	42	64.5	61.1	probiotics (Lactobacillus acidophilus, Lactobacillus rhamnosus, Lactobacillus paracasei, and Bifidobacterium lactis)/12 g/d, 5 preoperative days and 14 postoperative days	Oral	i, k	4
Kotzampassi et al. (2015) [56]	Greece	CRC surgery patients	84	80	65.9 ±11.5	66.4 ± 11.9	probiotics (Lactobacillus acidophilus LA-5, Lactobacillus plantarum, Bififidobacterium lactis BB-12, and Saccharomyces boulardii)/2 capsules/d, on the day of operation and 14 postoperative days	Oral/ EN	h, i, j, k	4
Sadahiro et al. (2014) [42]	Japan	CRC surgery patients	100	95	67 ± 9	66 ± 12	probiotics (Bifidobacteria)/9 tablets/d, 7 preoperative days and 5 postoperative days to 15 postoperative days	Oral	i, j	4
Xu et al. (2019) [43]	China	CRC surgery patients	30	30	61.03 ± 15.28	62.35 ± 13.71	probiotics (bifidus-triple viable preparation)/NA,7 preoperative days	Oral	a, i	4
Polakowski et al. (2019) [24]	Brazil	CRC surgery patients	36	37	60.9 ± 6.7	58.9 ± 6.3	probiotics (fructooligosaccharide, Lactobacillus acidophilus NCFM, L. rhamnosus HN001, L. casei LPC-37, and Bififidobacterium lactis HN019)/2 times/d, 7 preoperative days	Oral	a, c, g	7
Mizuta et al.(2016) [44]	Japan	CRC surgery patients	31	29	68.9 ± 10.4	71.2 ± 9.5	probiotics (B. longum)/2 g/d, 7–14 preoperative days and 14 postoperative days	Oral	c, d, g, i, j	7
Komatsu et al. (2016) [45]	Japan	CRC surgery patients	168	194	66.7 ± 11.6	67.7 ± 10.7	probiotics (Lactobacillus casei, galactooligosaccharides, and Bifidobacterium breve)/NA, 7–11 preoperative days and 2–7 postoperative days	Oral	i, j	5
Radvar et al. (2020) [46]	Iran	CRC chemoradiotherapy patients	23	23	57.58 ± 12.78	62.89 ± 13.93	probiotics (Lactobacillus casei PXN 37, Lactobacillus rhamnosus PXN 54, Streptococcus thermophilus PXN 66, Bififidobacteriumbreve PXN 25, Lactobacillus acidophilus PXN 35, Bififidobacteriumlongum PXN 30, Lactobacillus bulgaricus PXN 39, Fructooligosaccharide, magnesium stearate, and hydroxypropyl methyl- cellulose)/2 times/d, 6 weeks	Oral	e, f	7
Xie et al. (2019) [47]	China	CRC surgery patients	66	69	62.62 ± 9.627	60.29 ± 9.54	probiotics (fructooligosaccharide, xylooligosaccharide, polydextrose, and resistant dextrin)/30 g/d, 7 preoperative days	Oral	d	6
Liu et al. (2015) [48]	China	CRC surgery patients	66	68	65.62 ± 18.18	60.16 ± 16.20	probiotics (Lactobacillus plantarum, Lactobacillus acidophilus-11, and Bifido-bacterium longum-88)/2 g/d, 6 preoperative days and 10 postoperative days	Oral	g, h, i, k	7
Liu et al. (2011) [49]	China	CRC surgery patient	50	50	65.3 ± 11.0	65.7 ± 9.9	probiotics (Lactobacillus plantarum, Lactobacillus acidophilus-11, and Bifido-bacterium longum-88)/2 g/d, 6 preoperative days and 10 postoperative days	Oral	g, h, i, k	7
Yang et al. (2016) [50]	China	CRC surgery patients	30	30	63.90 ± 12.25	62.17 ± 11.06	probiotics (Bifidobacterium longum, Lactobacillus acidophilus, and Enterococcus faecalis)/6 g/d, 5 preoperative days and 7 postoperative days	Oral	d, g, h, i, j, k	6
Szefel et al. (2022) [57]	Poland	CRC surgery patients	28	37	68.9 ± 10.9	68.7 ± 9.3	arginine/20 capsules/d, 9 preoperative days	Oral	a	7
Braga et al. (2002) [27]	Italy	CRC surgery patients	50	50	63.0 ± 8.1	62.2 ± 10.4	arginine (12.5 g/L) + omega 3 fatty acid (3.3 g/L)/1 L/d, 5 preoperative days	Oral/ EN	c, g, h, i, j	5
Rotovnik et al. (2011) [58]	Slovenia	CRC chemoradiotherapy patients	14	19	60.5 ± 14.2	63.6 ± 10.12	glutamine/30 g/d, 5 weeks	Oral	a, c	7
Oguz et al. (2007) [51]	Turkey	CRC surgery patients	57	52	52 ± 12	57 ± 17	glutamine/1 g/kg/day, 5 preoperative days and 5 postoperative days	PN	g, h, i, j,	4
Rotovnik et al. (2017) [59]	Slovenia	CRC chemoradiotherapy patients	33	40	60.8 ±11.9	61.4 ± 9.9	glutamine/30 g/d, 5 weeks	Oral	c	6
Cui et al. (2014) [22]	China	CRC surgery patients	20	20	55 ± 10.8	56 ± 10.7	glutamine/0.5 g/kg, 1 preoperative day and the day of surgery	PN	b, g	6
Decker et al. (1999) [60]	German	CRC chemotherapy patients	12	12	56.1 ± 9.6	58.4 ± 7.2	glutamine/14 ± 22 g/d, 18 days	PN	e	4
Zaharuddin et al. (2019) [20]	Malaysia	CRC surgery patients	27	25	NR	NR	probiotics (Lactobacillus and Bifidobacteria)/2 times/d, 6 postoperative months	Oral	b, c	5
Morlion et al. (1998) [61]	German	CRC surgery patients	15	13	67.1 ± 10.7	68.2.1 ± 12.5	glutamine/0.3 g/kg/d/5 postoperative days	PN	g	6
Silva et al. (2012) [64]	Brazil	CRC chemotherapy patients	11	12	50.1 ± 8.2	54.3 ± 9.3	omega 3 fatty acid/0.6 g/d/9 weeks	Oral	a, c, d, e, f	4
Zhu et al. (2012) [52]	China	CRC surgery patients	29	28	69.8 ± 10.5	70.8 ± 6.4	omega 3 fatty acid/0.2 g/kg/d/7 postoperative days	PN	b, c, g, h, i	7

Note: NR, not report; PL, placebo; PN, parenteral nutrition; EN, enteral nutrition; CRC, Colorectal cancer. a, C-reactive protein; b, tumor necrosis factor- α; c, interleukin-6; d, albumin; e, weight; f, body mass index; g, the length of hospital stays; h, urinary infections; i, wound infections; j, anastomotic leaks; k, pneumonia.

## Data Availability

The data that support the findings of this study are available from the corresponding author upon reasonable request.

## Supplementary Materials

The following supporting information can be downloaded at https://www.mdpi.com/article/10.3390/nu15122772/s1. Supplementary Table S1: Search strategy to find potentially relevant articles for inclusion in the network meta-analysis. Supplementary Table S2 League Table: Nutritional supplements network meta-analysis results for C-reactive protein (CRP, mg/L). Supplementary Table S3 League Table: Nutritional supplements network meta-analysis results for nutritional indicators. (A) Albumin (Alb, g/L); (B) weight (kg); (C) body mass index (BMI, kg/m^2^). Supplementary Table S4 League Table: Nutritional supplements network meta-analysis results for clinical outcomes. (A) The incidence of urinary tract infections (UTI); (B) the incidence of anastomotic leaks (AL). Supplementary Table S5: SUCRA values of nutritional supplements according to inflammatory indicators. Supplementary Table S6: SUCRA values of nutritional supplements according to nutritional indicators. Supplementary Table S7: SUCRA values of nutritional supplements according to clinical outcomes. Supplementary Figure S1: Network plots for C-reactive protein (CRP, mg/L). Supplementary Figure S2: Network plots for nutritional indicators. (A) Albumin (Alb, g/L); (B) weight (kg); (C) body mass index (BMI, kg/m^2^). Supplementary Figure S3: Network plots for clinical outcomes. (A) The incidence of urinary tract infections; (B) the incidence of anastomotic leaks. Supplementary Figure S4 Funnel Plot: Funnel plot for the network of (A) tumor necrosis factor- α (TNF-α); (B) interleukin-6 (IL-6); (C) C-reactive protein (CRP); (D) the length of hospital stay (LOH); (E) the incidence of urinary tract infections (UTI); (F) the incidence of wound infections (WI); (G) the incidence of anastomotic leaks (AL); (H) the incidence of pneumonia.
