# Supplementary material for: Comparative Effects of Different Nutritional Supplements on Inflammation, Nutritional Status, and Clinical Outcomes in Colorectal Cancer Patients: A Systematic Review and Network Meta-Analysis
Source: Nutrients. 2023 Jun 16;15(12):2772. doi: 10.3390/nu15122772 (PMC10305393; doi:10.3390/nu15122772)
Supplement: Supplementary file 1 [file nutrients-15-02772-s001.zip › nutrients-2415434-SI.pdf]

Supplementary Tables

Supplementary Table S1. Search strategy to find potentially relevant articles for inclusion in the network meta-analysis.

Database	Search strategies
PubMed	#1 "Colorectal Neoplasms"[Mesh]
	#2 "Colorectal Neoplasms"[Title/Abstract] OR "Colorectal Cancer"[Title/Abstract] OR "Colorectal Cancers"[Title/Abstract] OR "Colorectal Carcinoma"[Title/Abstract] OR "Colorectal Carcinomas"[Title/Abstract] OR "Colorectal Neoplasm"[Title/Abstract] OR "Colorectal Tumor"[Title/Abstract] OR "Colorectal Tumors"[Title/Abstract]
	#3 #1 OR #2
	#4 "Glutamine"[Mesh]
	#5 "Glutamine"[Title/Abstract] OR "L-Glutamine"[Title/Abstract] OR "L Glutamine"[Title/Abstract] OR "D-Glutamine"[Title/Abstract] OR "D Glutamine"[Title/Abstract]
	#6 #4 OR #5
	#7 "Arginine"[Mesh]
	#8 "Arginine"[Title/Abstract] OR "DL-Arginine Acetate, Monohydrate"[Title/Abstract] OR "L-Arginine"[Title/Abstract] OR "L-Isomer Arginine"[Title/Abstract] OR "L Arginine"[Title/Abstract] OR "Monohydrate DL-Arginine Acetate"[Title/Abstract]
	#9 #7 OR #8
	#10 "Fatty Acids, Omega-3"[Mesh]
	#11 "Fatty Acids, Omega-3"[Title/Abstract] OR "Fatty Acid, Omega-3"[Title/Abstract] OR "N 3 Fatty Acid"[Title/Abstract] OR "n 3 Fatty Acids"[Title/Abstract] OR "n 3 Oil"[Title/Abstract] OR "n 3 Oils"[Title/Abstract] OR "n 3 Polyunsaturated Fatty Acid"[Title/Abstract] OR "n 3 PUFA"[Title/Abstract] OR "N-3 Fatty Acid"[Title/Abstract] OR "n3 Fatty Acid"[Title/Abstract] OR "n-3 Fatty Acids"[Title/Abstract] OR "n-3 Oil"[Title/Abstract] OR "n-3 Oils"[Title/Abstract] OR "n3 Polyunsaturated Fatty Acid"[Title/Abstract] OR "n-3 Polyunsaturated Fatty Acid"[Title/Abstract] OR "n-3 PUFA"[Title/Abstract] OR "n3 PUFA"[Title/Abstract] OR "Omega 3 Fatty Acid"[Title/Abstract] OR "Omega 3 Fatty Acids"[Title/Abstract] OR "Omega-3 Fatty Acid"[Title/Abstract] OR "Omega-3 Fatty Acids"[Title/Abstract] OR "n3 Oils"[Title/Abstract] OR "n3 Oil"[Title/Abstract]
	#12 #10 OR #11
	#13 "Probiotics"[Mesh]
	#14 "Probiotics"[Title/Abstract] OR "Probiotic"[Title/Abstract]
	#15 #13 OR #14
	#16 "Vitamin D"[Mesh]
	#17 "Vitamin D"[Title/Abstract] OR "25-OH-D"[Title/Abstract] OR "25-hyroxy Vitamin D"[Title/Abstract] OR "1, 25-dihyroxy Vitamin D"[Title/Abstract] OR "cholecalciferol"[Title/Abstract] OR "calcidiol"[Title/Abstract] OR

	"calcitriol"[Title/Abstract] OR "hydroxycholecalciferols"[Title/Abstract] OR "25-hydroxy Vitamin D3"[Title/Abstract]
	#18 #16 OR #17
	#19 #6 OR #9 OR #12 OR #15 OR #18
	#20 #3 AND #19
Web of Science	#1 Colorectal Neoplasms (Topic) OR Colorectal Cancer (Topic) OR Colorectal Cancers (Topic) OR Colorectal Carcinoma (Topic) OR Colorectal Carcinomas (Topic) OR Colorectal Neoplasm (Topic) OR Colorectal Tumor (Topic) OR Colorectal Tumors (Topic)
	#2 Glutamine (Topic) OR L-Glutamine (Topic) OR L Glutamine (Topic) OR D-Glutamine (Topic) OR D Glutamine (Topic)
	#3 Arginine (Topic) OR DL-Arginine Acetate, Monohydrate (Topic) OR L-Arginine (Topic) OR L-Isomer Arginine (Topic) OR L Arginine (Topic) OR Monohydrate DL-Arginine Acetate (Topic)
	#4 Fatty Acids, Omega-3 (Topic) OR Fatty Acid, Omega-3 (Topic) OR N 3 Fatty Acid (Topic) OR n 3 Fatty Acids (Topic) OR n 3 Oil (Topic) OR n 3 Oils (Topic) OR n 3 Polyunsaturated Fatty Acid (Topic) OR n 3 PUFA (Topic) OR N-3 Fatty Acid (Topic) OR n3 Fatty Acid (Topic) OR n-3 Fatty Acids (Topic) OR n-3 Oil (Topic) OR n-3 Oils (Topic) OR n3 Polyunsaturated Fatty Acid (Topic) OR n-3 Polyunsaturated Fatty Acid (Topic) OR n-3 PUFA (Topic) OR n3 PUFA (Topic) OR Omega 3 Fatty Acid (Topic) OR Omega 3 Fatty Acids (Topic) OR Omega-3 Fatty Acid (Topic) OR Omega-3 Fatty Acids (Topic) OR n3 Oils (Topic) OR n3 Oil (Topic)
	#5 Probiotics (Topic) OR Probiotic (Topic)
	#6 Vitamin D (Topic) OR 25-OH-D (Topic) OR 25-hyroxy Vitamin D (Topic) OR 1, 25-dihyroxy Vitamin D (Topic) OR cholecalciferol (Topic) OR calcidiol (Topic) OR calcitriol (Topic) OR hydroxycholecalciferols (Topic) OR 25-hydroxy Vitamin D_3 (Topic)
	#7 #6 OR #5 OR #4 OR #3 OR #2
	#8 #7 AND #1
Embase	#1 'colorectal cancer'/exp OR 'colorectal tumor'/exp
	#2 'colorectal cancer':ti,ab OR 'colo-rectal cancer':ti,ab OR 'colo-rectal carcinogenesis':ti,ab OR 'colo-rectal malignancies':ti,ab OR 'colo-rectal malignancy':ti,ab OR 'colorectal cancerogenesis':ti,ab OR 'colorectal carcinogenesis':ti,ab OR 'colorectal malignancies':ti,ab OR 'colorectal malignancy':ti,ab OR 'recto-colonic cancer':ti,ab OR 'rectocolonic cancer':ti,ab OR 'colorectal tumor':ti,ab OR 'colorectal neoplasia':ti,ab OR 'colorectal neoplasm':ti,ab OR 'colorectal neoplasms':ti,ab OR 'colorectal tumorigenesis':ti,ab OR 'colorectal tumour':ti,ab OR 'neoplastic colorectal':ti,ab
	#3 #1 OR #2
	#4 'glutamine'/exp
	#5 'glutamine':ti,ab OR '2 aminoglutaramic acid':ti,ab OR '2, 5 diamino 5 oxopentanoic acid':ti,ab OR 'acutil':ti,ab OR 'adamin g':ti,ab OR 'endari':ti,ab OR 'glumin':ti,ab OR 'glutamic acid 5 amide':ti,ab OR 'glutamic acid monoamide':ti,ab OR 'glutamin':ti,ab OR 'levoglutamide':ti,ab OR 'levoglutamine':ti,ab OR 'nutrestore':ti,ab OR 'xyndari':ti,ab OR '2 amino 4

	carbamoylbutanoic acid':ti,ab
	#6 #4 OR #5
	#7 'arginine'/exp
	#8 'arginine':ti,ab OR 'arg':ti,ab OR 'arginin':ti,ab OR 'arginyl residue':ti,ab OR 'argivene':ti,ab OR 'bioarginina':ti,ab OR 'l a 60 45':ti,ab OR 'l arginine':ti,ab OR 'r gene 10':ti,ab OR 'r-gene 10':ti,ab OR '2 amino 5 guanidinovaleric acid':ti,ab
	#9 #7 OR #8
	#10 'omega 3 fatty acid'/exp
	#11 'omega 3 fatty acid':ti,ab OR 'eicosa e':ti,ab OR 'eicosapen':ti,ab OR 'epaisdin':ti,ab OR 'epanova':ti,ab OR 'n 3 fatty acid':ti,ab OR 'n 3 polyunsaturated fatty acid':ti,ab OR 'omega 3*':ti,ab OR 'omega forte':ti,ab OR 'omega-3-carboxylic acids':ti,ab OR 'sakana':ti,ab OR 'sanhelios omega 3':ti,ab OR 'bilantin omega':ti,ab
	#12 #10 OR #11
	#13 'probiotic agent'/exp
	#14 'probiotic agent':ti,ab OR 'probiotic':ti,ab OR 'probiotics':ti,ab
	#15 #13 OR #14
	#16 'vitamin d'/exp
	#17 'vitamin d':ti,ab OR '25-oh-d':ti,ab OR '25-hyroxy vitamin d':ti,ab OR '1, 25-dihyroxy vitamin d':ti,ab OR 'cholecalciferol':ti,ab OR 'calcidiol':ti,ab OR 'calcitriol':ti,ab OR 'hydroxycholecalciferols':ti,ab OR '25-hydroxy vitamin d_3':ti,ab
	#18 #16 OR #17
	#19 #6 OR #9 OR #12 OR #15 OR #18
	#20 #3 AND #19
The Cochrane Library	#1 MeSH descriptor: [Colorectal Neoplasms] explode all trees
	#2 (Colorectal Neoplasms):ti,ab,kw OR (Colorectal Cancer):ti,ab,kw OR (Colorectal Cancers):ti,ab,kw OR (Colorectal Carcinoma):ti,ab,kw OR (Colorectal Carcinomas):ti,ab,kw OR (Colorectal Neoplasm):ti,ab,kw OR (Colorectal Tumor):ti,ab,kw OR (Colorectal Tumors):ti,ab,kw
	#3 #1 OR #2
	#4 MeSH descriptor: [Glutamine] explode all trees
	#5 (Glutamine):ti,ab,kw OR (L-Glutamine):ti,ab,kw OR (L Glutamine):ti,ab,kw OR (D-Glutamine):ti,ab,kw OR (D Glutamine):ti,ab,kw
	#6 #4 OR #5
	#7 MeSH descriptor: [Arginine] explode all trees
	#8 (Arginine):ti,ab,kw OR (DL-Arginine Acetate, Monohydrate):ti,ab,kw OR (L-Arginine):ti,ab,kw OR (L-Isomer Arginine):ti,ab,kw OR (L Arginine):ti,ab,kw OR (Monohydrate DL-Arginine Acetate):ti,ab,kw

	#9 #7 OR #8
	#10 MeSH descriptor: [Fatty Acids, Omega-3] explode all trees
	#11 (Fatty Acids, Omega-3):ti,ab,kw OR (Fatty Acid, Omega-3):ti,ab,kw OR (N 3 Fatty Acid):ti,ab,kw OR (n 3 Fatty Acids):ti,ab,kw OR (n 3 Oil):ti,ab,kw OR (n 3 Oils):ti,ab,kw OR (n 3 Polyunsaturated Fatty Acid):ti,ab,kw OR (n 3 PUFA):ti,ab,kw OR (N-3 Fatty Acid):ti,ab,kw OR (n3 Fatty Acid):ti,ab,kw OR (n-3 Fatty Acids):ti,ab,kw OR (n-3 Oil):ti,ab,kw OR (n-3 Oils):ti,ab,kw OR (n3 Polyunsaturated Fatty Acid):ti,ab,kw OR (n-3 Polyunsaturated Fatty Acid):ti,ab,kw OR (n-3 PUFA):ti,ab,kw OR (n3 PUFA):ti,ab,kw OR (Omega 3 Fatty Acid):ti,ab,kw OR (Omega 3 Fatty Acids):ti,ab,kw OR (Omega-3 Fatty Acid):ti,ab,kw OR (Omega-3 Fatty Acids):ti,ab,kw OR (n3 Oils):ti,ab,kw OR (n3 Oil):ti,ab,kw
	#12 #10 OR #11
	#13 MeSH descriptor: [Probiotics] explode all trees
	#14 (Probiotics):ti,ab,kw OR (Probiotic):ti,ab,kw
	#15 #13 OR #14
	#16 MeSH descriptor: [Vitamin D] explode all trees
	#17 (Vitamin D):ti,ab,kw OR (cholecalciferol):ti,ab,kw OR (calcidiol):ti,ab,kw OR (calcitriol):ti,ab,kw OR (hydroxycholecalciferols):ti,ab,kw
	#18 #16 OR #17
	#19 #6 OR #9 OR #12 OR #15 OR #18
	#19 AND #3

Supplementary Table S2 League table: Nutritional supplements network meta-analysis results for C-reactive protein (CRP, mg/l).

vitamind							
−15.11 (−69.87, 39.99)	omega3 + vitamind						
−27.18 (−99.47, 42.44)	−12 (−78.55, 51.2)	arginine					
−34.39 (−88.54, 20.71)	−19.27 (−65.28, 26.79)	−7.24 (−55.66, 45)	omega3				
−35.17 (−96.4, 27.94)	−20.29 (−74.16, 35.97)	−8.43 (−58.19, 48.28)	−1.19 (−37.36, 37.59)	probiotics			
−43.12 (−111.97, 23.29)	−27.95 (−91.03, 31.16)	−15.87 (−75.78, 43.6)	−8.23 (−56.99, 35.48)	−6.93 (−60.46, 38.4)	glutamine		
−47.1 (−115.36, 18.52)	−31.92 (−94.27, 26.95)	−19.77 (−79.31, 39.15)	−11.9 (−60.53, 31)	−10.45 (−63.78, 33.78)	−3.95 (−59.46, 52.04)	omega3 + probiotics	
−46.34 (−102.68, 8.33)	−31.26 (−79.47, 14.38)	−19 (−63.66, 25.48)	−11.47 (−37.21, 9.53)	−10.04 (−43.3, 15.11)	−3.08 (−42.83, 36.69)	0.83 (−38.6, 40.09)	placebo

Notes: estimates are presented as mean difference (MD) and 95% credible intervals (CrI; in parentheses). Comparisons between treatments should be read from left to right. The estimate of supplementation effectiveness is located at the intersection of the column-defining supplementation and the

row-defining supplementation. Significant results were presented in bold. Combined with omega-3 fatty acids and probiotics: omega3 + probiotics; combined with omega-3 fatty acids and vtamin D: omega3 + vitmind; omega-3 fatty acids: omega3; vitamin D: vitamind.

Supplementary Table S3 League table: Nutritional supplements network meta-analysis results for nutritional indicators. (A) albumin (Alb, g/l); (B) weight (kg); (C) body mass index (BMI, kg/m²).

(A)

omega3 + vitamind				
2.01 (−9.21, 13.18)	vitamind			
2.57 (−7.3, 12.88)	0.56 (−9.6, 11.21)	omega3		
4.37 (−7.28, 16.84)	2.37 (−9.53, 15.14)	1.78 (−7.03, 11.05)	probiotics	
5.3 (−4.51, 15.96)	3.3 (−6.89, 14.25)	2.7 (−3.47, 9.33)	0.92 (−5.43, 7.31)	placebo

Notes: estimates are presented as mean difference (MD) and 95% credible intervals (CrI; in parentheses). Comparisons between treatments should be read from left to right. The estimate of supplementation effectiveness is located at the intersection of the column-defining supplementation and the row-defining supplementation. Significant results were presented in bold. Combined with omega-3 fatty acids and vtamin D: omega3 + vitmind; omega-3 fatty acids: omega3; vitamin D: vitamind.

(B)

omega3 + vitamind						
0.45 (−5.87, 6.83)	omega3					
1.01 (−6.27, 8.36)	0.54 (−6.21, 7.27)	vitamind				
1.93 (−10.23, 13.92)	1.5 (−9.29, 12.1)	0.93 (−11.56, 13.16)	glutamine			
2.2 (−6.03, 10.56)	1.76 (−4.3, 7.91)	1.22 (−7.29, 9.86)	0.31 (−10.99, 11.77)	omega3 + probiotics		
3.08 (−7.05, 13.04)	2.58 (−5.8, 10.95)	2.04 (−8.23, 12.49)	1.15 (−11.68, 14.02)	0.83 (−8.45, 10.11)	probiotics	
2.44 (−4.01, 8.94)	1.95 (−1.3, 5.4)	1.45 (−5.38, 8.28)	0.5 (−9.58, 10.72)	0.21 (−4.91, 5.32)	−0.61 (−8.34, 7.16)	placebo

Notes: estimates are presented as mean difference (MD) and 95% credible intervals (CrI; in parentheses). Comparisons between treatments should be read from left to right. The estimate of supplementation effectiveness is located at the intersection of the column-defining supplementation and the row-defining supplementation. Significant results were presented in bold. Combined with omega-3 fatty acids and probiotics: omega3 + probiotics; combined with omega-3 fatty acids and vtamin D: omega3 + vitmind; omega-3 fatty acids: omega3; vitamin D: vitamind.

(C)

omega3 + vitamind					
0.11 (−2.05, 2.27)	omega3				
0.2 (−2.27, 2.69)	0.09 (−2.12, 2.31)	vitamind			
0.85 (−4.62, 6.43)	0.71 (−4.45, 6.03)	0.66 (−4.87, 6.28)	omega3 + probiotics		
0.7 (−2.83, 4.28)	0.6 (−2.43, 3.68)	0.52 (−3.09, 4.11)	−0.12 (−6, 5.54)	probiotics	
0.9 (−1.27, 3.1)	0.78 (−0.41, 2.05)	0.7 (−1.53, 2.96)	0.07 (−5.1, 5.04)	0.19 (−2.63, 2.97)	placebo

Notes: estimates are presented as mean difference (MD) and 95% credible intervals (CrI; in parentheses). Comparisons between treatments should be read from left to right. The estimate of supplementation effectiveness is located at the intersection of the column-defining supplementation and the row-defining supplementation. Significant results were presented in bold. Combined with omega-3 fatty acids and probiotics: omega3 + probiotics; combined with omega-3 fatty acids and vtamin D: omega3 + vitmind; omega-3 fatty acids: omega3; vitamin D: vitamind.

Supplementary Table S4 League table: Nutritional supplements network meta-analysis results for clinical outcomes. (A) the incidence of urinary tract infections (UTI); (B) the incidence of anastomotic leaks (AL).

(A)

probiotics				
0.51 (0.01, 23.92)	omega3 + arginine			
0.31 (0.05, 1.6)	0.62 (0.02, 18.84)	placebo		
0.21 (0, 8.98)	0.41 (0, 53.29)	0.68 (0.02, 20.47)	glutamine	
0.14 (0.01, 2.56)	0.27 (0, 18.63)	0.45 (0.04, 5.11)	0.65 (0.01, 49.99)	omega3

Notes: estimates are presented as risk ratio (RR) and 95% credible intervals (CrI; in parentheses). Comparisons between treatments should be read from left to right. The estimate of supplementation effectiveness is located at the intersection of the column-defining supplementation and the row-defining supplementation. . Combined with omega-3 fatty acids and arginine: omega3 + arginine; omega-3 fatty acids: omega3.

(B)

glutamine				
0.35 (0, 21.57)	omega3 + arginine			
0.26 (0, 7.63)	0.72 (0.05, 14.14)	probiotics		
0.17 (0, 3.66)	0.47 (0.03, 6.4)	0.66 (0.2, 1.54)	placebo	
0.04 (0, 1.27)	0.11 (0, 2.31)	0.15 (0.01, 1.08)	0.24 (0.02, 1.44)	omega3

Notes: estimates are presented as risk ratio (RR) and 95% credible intervals (CrI; in parentheses). Comparisons between treatments should be read from left to right. The estimate of supplementation effectiveness is located at the intersection of the column-defining supplementation and the

row-defining supplementation. . Combined with omega-3 fatty acids and arginine: omega3 + arginine; omega-3 fatty acids: omega3.

Supplementary Table S5 SUCRA values of nutritional supplements according to inflammatory indicators.

Outcomes	Rank	1	2	3	4	5	6	7	8
TNF-α	Supplement	Glutamine	Probiotics	Omega-3+vitamin D	Vitamin D	Omega-3	Omega-3+Probiotics	Placebo	
	SUCRA (%)	99.9	83.3	51.0	42.9	31.1	28.1	13.7	
IL-6	Supplement	Omega-3+Arginine	Probiotics	Omega-3+Probiotics	Vitamin D	Omega-3+Vitamin D	Glutamine	Omega-3	Placebo
	SUCRA (%)	98.6	75.8	44.5	41.9	40.4	35.7	32.4	30.7
CRP	Supplement	Vitamin D	Omega-3+ Vitamin D	Arginine	Omega-3	Probiotics	Glutamine	Omega-3+Probiotics	Placebo
	SUCRA (%)	87.2	74.6	60.5	51.4	49.3	32.7	23.3	20.9

Note: omega-3 fatty acids: omega-3.

Supplementary Table S6 SUCRA values of nutritional supplements according to nutritional indicators.

Outcomes	Rank	1	2	3	4	5	6	7
Albumin	Supplement	Omega-3+Vitamin D	Vitamin D	Omega-3	Probiotics	Placebo		
	SUCRA (%)	76.9	58.0	56.1	37.0	22.1		
Weight	Supplement	Omega-3+Vitamin D	Omega-3	Vitamin D	Glutamine	Omega-3+Probiotics	Probiotics	Placebo
	SUCRA (%)	66.6	66.5	55.3	47.4	42.1	36.3	35.7
BMI	Supplement	Omega-3+Vitamin D	Omega-3	Vitamin D	Omega-3+Probiotics	Probiotics	Placebo	
	SUCRA (%)	63.6	63.4	56.9	43.7	42.8	29.6	

Note: omega-3 fatty acids: omega-3.

Supplementary Table S7 SUCRA values of nutritional supplements according to clinical outcomes.

Outcomes	Rank	1	2	3	4	5	6	7
the length of hospital stay	Supplement	Glutamine	Omega-3	Omega-3+Glutamine	Omega3+Arginine	Probiotics	Placebo	
	SUCRA (%)	78.7	71.1	58.5	56.1	31.5	4.0	
the incidence of urinary tract infection	Supplement	Probiotics	Omega-3+Arginine	Placebo	Glutamine	Omega-3		
	SUCRA (%)	83.5	59.9	45.3	37.5	23.8		

the incidence of wound infections	Supplement	Glutamine	Probiotics	Omega-3+Arginine	Placebo	Omega-3
	SUCRA (%)	94.5	60.6	54.6	21.0	19.4
the incidence of anastomotic leaks	Supplement	Glutamine	Omega-3+Arginine	Probiotics	Placebo	Omega-3
	SUCRA (%)	84.1	64.5	60.2	36.6	4.5
the incidence of pneumonia	Supplement	Probiotics	Glutamine	Placebo	Omega-3	
	SUCRA (%)	82.0	69.1	34.5	14.5	

Note: omega-3 fatty acids: omega-3.

Supplementary Figures

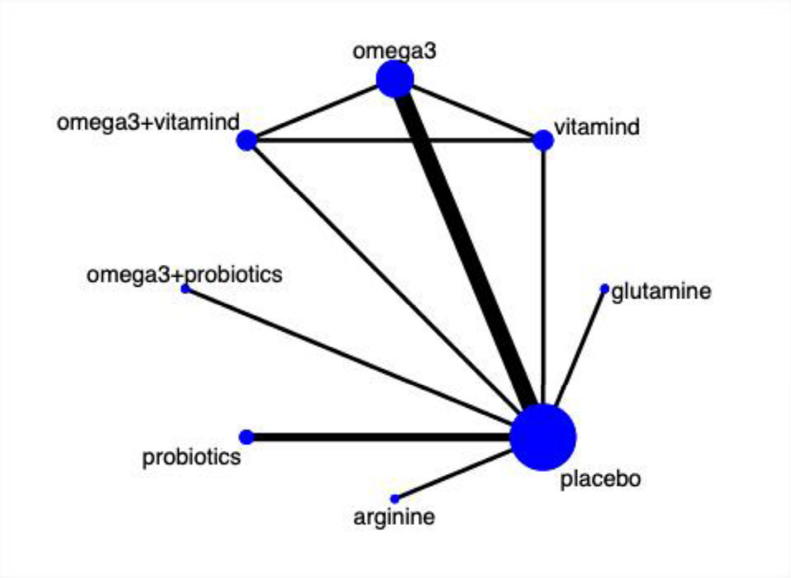

Supplementary Figure S1 Network plots for C-reactive protein (CRP, mg/l).

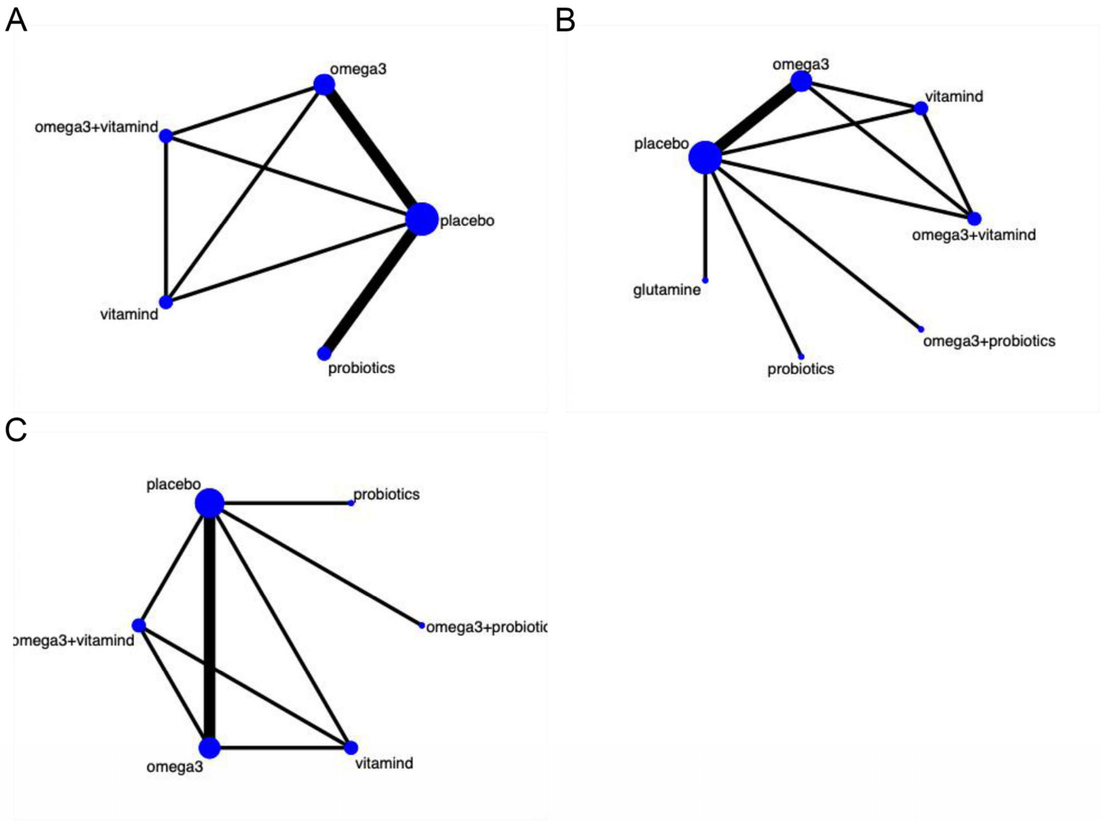

Supplementary Figure S2 Network plots for nutritional indicators. (A) albumin (Alb, g/l); (B) weight (kg); (C) body mass index (BMI, kg/m²).

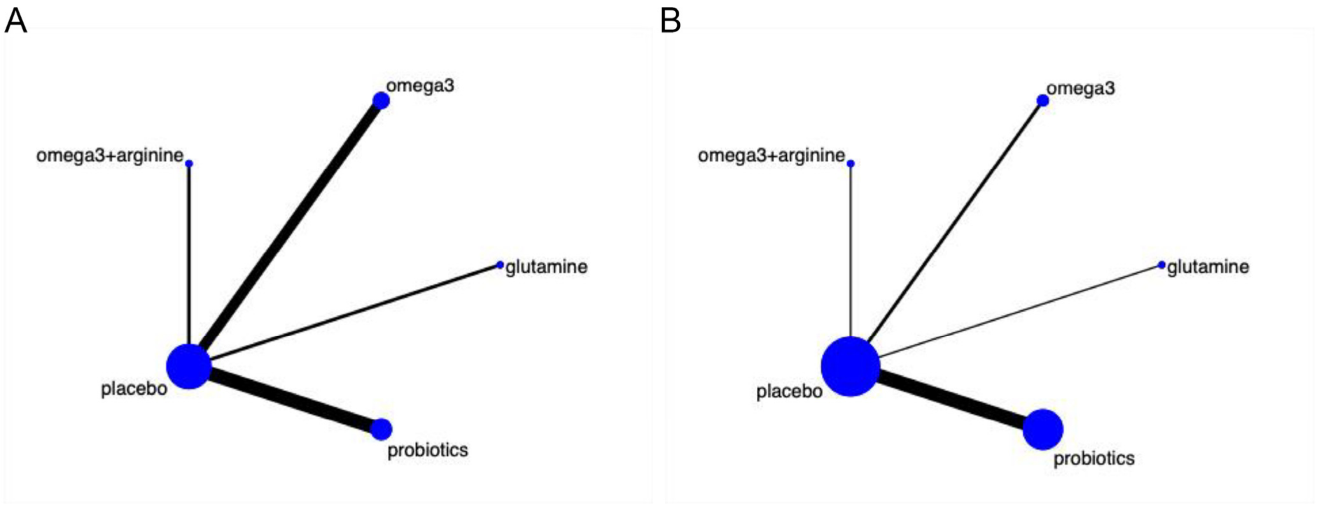

Supplementary Figure S3 Network plots for clinical outcomes. (A) the incidence of urinary tract infections; (B) the incidence of anastomotic leaks.

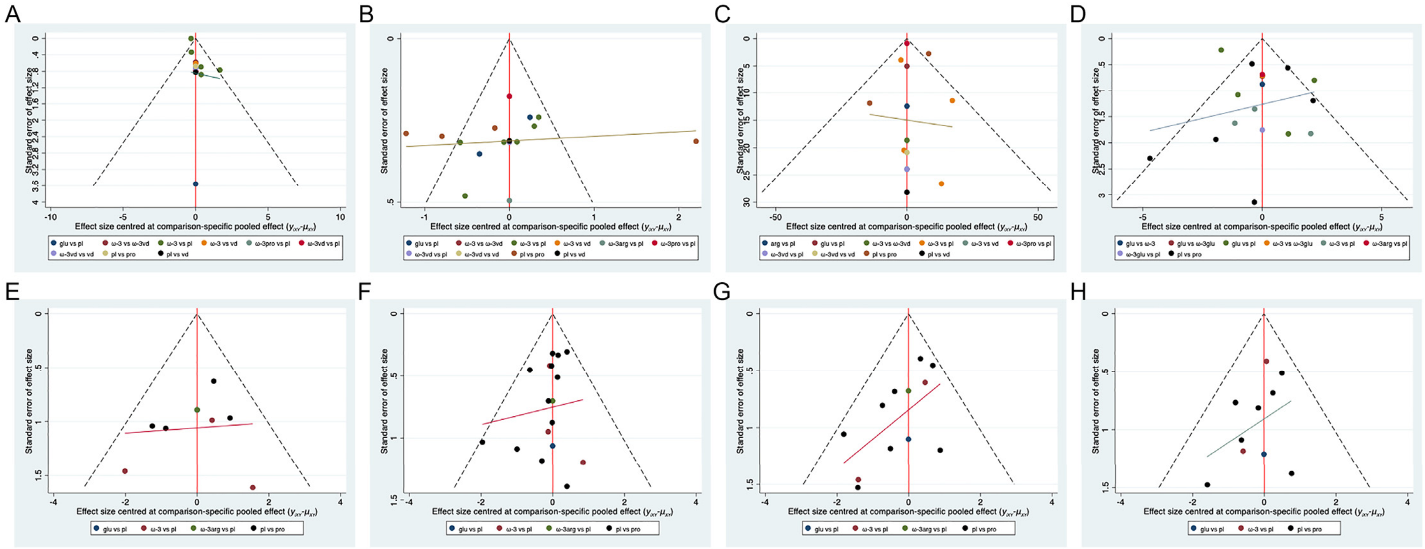

Supplementary Figure S4 Funnel plot. Funnel plot for the network of (A) tumor necrosis factor- α (TNF-α); (B) Interleukin-6 (IL-6); (C) C-reactive protein (CRP); (D) the length of hospital stay (LOH); (E) the incidence of urinary tract infections (UTI); (F) the incidence of wound infections (WI); (G) the incidence of anastomotic leaks (AL); (H) the incidence of pneumonia.
